# Phase Angle and Postoperative Complications in a Model of Immunonutrition in Patients with Pancreatic Cancer

**DOI:** 10.3390/nu15204328

**Published:** 2023-10-11

**Authors:** Magdalena Boćkowska, Przemysław Kostro, Zbigniew Krzysztof Kamocki

**Affiliations:** Second Department of General and Gastroenterological Surgery, Medical University of Bialystok, M. Sklodowskiej-Curie Street 24a, 15-276 Bialystok, Poland

**Keywords:** immunonutrition, phase angle, complications, pancreatic cancer

## Abstract

Background: The aim of this study was to determine the influence of our own model of immunonutrition on phase angle and postoperative complications. Our goal was to establish modern prehabilitation procedures for patients operated on for pancreatic cancer. Methods: Patients with pancreatic cancer who qualified for surgical treatment were divided into two groups. Group I (20 patients; 12 with pancreatic head cancer, 8 with pancreatic tail/body cancer) was given immunonutrition (Impact Oral 3× a day, 237 mL, for 5 days before surgery, and after surgery for an average of 3.5 days). Group II (20 patients; 12 with pancreatic head cancer, 8 with pancreatic tail/body cancer) did not receive immunonutrition. Body weight, body mass index and phase angle were assessed on admission to the hospital, after preoperative immunonutrition, on the third and eighth postoperative days. C-reactive protein and Interleukin-1 α were measured on admission to the hospital, after preoperative immunonutrition, on the eighth postoperative day. Postsurgical complications were assessed via Clavien–Dindo classification. Results: On admission to the hospital, the phase angle was 5.0° (4.70–5.85) in Group I and 5.1° (5.00–6.25) in Group II. After 5 days of using preoperative immunonutrition, it increased statistically significantly (*p* < 0.02) to 5.35°. In Group I, on the third day after surgery, it decreased statistically significantly (*p* < 0.001) to 4.65°, and then, increased to 4.85° on the eighth day. In Group II, statistically significant decreases in the phase angle were observed on the third (4.5°; *p* < 0.002) and eighth (4.55°; *p* < 0.008) days after surgery. A statistically significant increase in CRP (86.6 mg/dL; *p* < 0.02) and IL-1α (18.5 pg/mL; *p* < 0.03) levels was observed on the eighth day after surgery in this group. In Group I, a statistically significant negative correlation (R −0.501106; *p* < 0.002) of the phase angle after 5 days of preoperative immunonutrition with postoperative complications was observed. Conclusions: This study used our own model of immunonutrition in patients undergoing surgery for pancreatic cancer. The applied model of perioperative IN improved the postoperative course of patients operated on due to pancreatic cancer. Fewer complications were observed in patients in the group receiving IN. Also, the PA value increased after the 5-day preoperative IN, and the use of perioperative IN improved the PA value on the eighth postoperative day compared to the group that did not receive IN. On this day, an increase in inflammatory parameters was also observed in the group that did not receive IN. In addition, PA correlated negatively with complications. The PA can be a useful tool to assess the effectiveness of the applied IN, and thus, to predict the occurrence of postoperative complications. Therefore, there is a further need for studies on larger groups of patients.

## 1. Introduction

Pancreatic cancer is one of the deadliest cancers in the world. In most countries, there is an upward trend in morbidity and mortality due to pancreatic cancer. Surgical treatment is still the main method of treatment; however, it is associated with a high risk of complications. In addition, patients with pancreatic cancer are characterized by an abnormal nutritional status. Rapidly progressing malnutrition occurs in as many as 85% of patients, which results in high morbidity in the perioperative period [1]. Pancreatic cancer induces an immunosuppressive inflammatory reaction. Improvement of the postoperative course is seen in perioperative immunonutrition (IN). In particular, arginine, omega-3 fatty acids and nucleotides have immunomodulating effects. During stress and catabolism, the demand for this ingredient increases dramatically, especially in critically ill patients. It is a substrate for the production of nitric oxide, whose role is, among other things, to influence wound healing [2,3,4,5]. According to studies, omega-3 fatty acids may reduce the length of hospital stay and the incidence of complications by modulating eicosanoids and cytokines [6]. Nucleotides improve the functioning of the immune system and accelerate the maturation of T lymphocytes, which facilitates the fight against bacterial infections [6].

According to the 2021 European Society for Clinical Nutrition and Metabolism (ESPEN) recommendations regarding patients with cancer of the upper gastrointestinal tract undergoing surgical treatment, it is recommended to use IN containing arginine, omega-3 fatty acids and nucleotides in the perioperative or at least postoperative period. Unfortunately, there are currently no clear recommendations regarding the amount and duration of IN in gastrointestinal cancers [7,8].

Electrical bioimpedance (BIA) is a method of assessing body composition, which, due to the non-invasiveness and painlessness of the procedure, and the simplicity of implementation, has been used in clinical practice. The BIA test is based on the difference in current conduction in the water and fat compartments of the body. It uses the measurement of impedance, i.e., the type of electrical resistance of tissues (composed of resistance and reactance) through which a low current (1 mA) is passed. BIA helps determine body components such as total body water (it can be divided into extracellular water and intracellular water), fat mass, fat-free mass and body cell mass, as well as the phase angle (PA). An increasingly appreciated parameter is the phase angle. The value of the PA is believed to correlate with cellular health. The PA is calculated using the arctangent value of the ratio of reactance to resistance. When alternating current flows through the human body, healthy cell membranes act as capacitors. They store electricity, causing a delay in its flow. This lag in the current that penetrates cell membranes and tissue interfaces creates a phase difference between the current and voltage, which is expressed as the PA [9,10]. A high value indicates well-nourished cells, while low values correlate with malnutrition. Research shows that low PA values are observed in patients with chronic diseases, HIV and oncology. Correlations have been observed with low muscle strength, skeletal muscle mass and patients’ quality of life. More and more attention is paid to its practical value in oncology [11,12].

Our goal is to establish modern prehabilitation procedures for patients operated on for pancreatic cancer.

The aim of this study was to determine the influence of our own model of IN on the PA and postoperative complications.

## 2. Materials and Methods

### 2.1. Subjects

The study included 57 patients admitted to the 2nd Department of General, Gastroenterological and Oncological Surgery of the University Clinical Hospital in Bialystok for the surgical treatment of pancreatic cancer between June 2021 and July 2023. Then, 40 patients were included in the study after confirmation of a malignant neoplasm in histopathological examination.

The study excluded 5 patients in whom pancreatic cancer was not confirmed histopathologically, 6 patients in whom non-resectable lesions were found intraoperatively, 3 patients with an implanted cardiac defibrillator and 3 patients who died in the early postoperative days.

### 2.2. Immunonutrition

Impact is a Food for Special Medical Purposes, produced by Nestle Health Science (Vevey, Switzerland), that is nutritionally complete. It contains immunomodulatory ingredients such as L-arginine, omega-3 fatty acids and nucleotides. Impact Oral (Cat. No. 123855531) is administered orally and Impact Enteral (Cat. No. 12371414) is administered enterally. Impact Oral in 100 mL contains 144 kcal; 7.6 g of protein, including 1.8 g of L-arginine; 0.6 g of omega-3 fatty acids; and 0.18 g of nucleotides. Impact Enteral in 100 mL contains 101 kcal; 5.6 g of protein, including 1.3 g of L-arginine; 0.33 g of omega-3 fatty acids; and 0.13 g of nucleotides.

The patients were divided into 2 groups. The patients in the first group were given IN for 5 days before the operation comprising Impact Oral 3 times a day (1 package of 237 mL). Subsequently, as part of early post-operative IN, Impact Oral or Impact Enteral was administered for an average of 3.5 days via a nasoenteric tube through continuous infusion using a mechanical pump. Patients in the second group did not receive IN. All subjects were fed orally and received a basic hospital diet before surgery.

### 2.3. Anthropometric Measurements and Body Composition Analysis

On admission to the hospital, in Group I after the end of preoperative IN, and then, in both groups on the third and eighth days after surgery, body weight was assessed using a scale available at the Clinic with an accuracy of 0.1 kg. The height of the patients was measured. BMI was calculated. Using bioelectrical impedance (Bodystat MDD 1500 analyzer by Bodystat, Douglas, Isle of Man), the PA values were evaluated. In order to standardize the tests, body composition analysis was performed in each patient on an empty stomach, at the patients’ beds in a supine position, by sticking electrodes to the right hand and foot.

### 2.4. Inflammation

On admission to the hospital, in Group I after the end of preoperative IN, and then, in both groups on the eighth day after surgery, C-reactive protein and Interleukin-1α were measured. Venous blood (5.5 mL) was collected on an empty stomach from all patients after overnight rest using the S-Monovette^®^ serum collection system (Sarstedt, Germany). Immediately after collection, the blood was centrifuged at 1500× *g* for 10 min at +4 °C (MPW 351, MPW Med. Instruments, Warsaw, Poland) to separate serum from erythrocytes. The top layer (serum) was then collected. Approximately 0.5 M butylated hydroxytoluene (20 μL/2 mL plasma or serum) was added to prevent sample oxidation. Until the selected parameters of inflammation were determined, all samples were stored at −80 °C.

Interleukins were assayed using a customized Bio-Plex Pro Human Cytokine Screening Panel, 48-Plex (#12007283) (Bio-Rad Laboratories, Life Science Group, Hercules, CA, USA).

### 2.5. Assessment of Postoperative Complications

A 30-day postoperative period was evaluated, with details of complications given according to the Clavien–Dindo classification.

### 2.6. Statistical Analysis

The results were analyzed in the statistical program STATISTICA 13.3. In order to assess the change in the phase angle value and the concentration of C-reactive protein and Interleukin-1α in the subsequent postoperative days, the non-parametric Wilcoxon test for related samples was used in each group. Spearman correlation was used to assess the relationship between the phase angle values in both groups and the occurrence of complications. Results with *p* < 0.05 were considered statistically significant.

### 2.7. Ethical Approval

All procedures followed were in accordance with the ethical standards of the institutional and national committee on human experimentation and the Helsinki Declaration. Written informed consent was obtained from all patients.

This study was approved by the Bioethical Commission at the Medical University of Bialystok, no. APK.002.279.2021. This research was funded by the Medical University of Bialystok (research number: SUB/1/DN/22/001/1137).

## 3. Results

### 3.1. Patient Characteristics

This study included 40 patients with histopathologically confirmed cancer of the pancreatic head or tail/body who had undergone surgical treatment, including 19 (47.5%) women and 21 (52.5%) men aged 46 to 85 (median 66; Q1–Q3 61.0–70.5). In 23 patients (82.5%), unintentional weight loss was observed in the last 6 months. It ranged from 1 kg (1.7%) to 30 kg (25.8%). The median was 8.0 kg, with a percentage of 9.9. In 18 patients (45%), it was greater than 10%. A total of 4 (10%) patients had a BMI below 20 kg/m^2^, 17 (42.5%) patients had a normal BMI, 14 (35%) patients were overweight, and 5 (12.5%) were obese at the 1st degree.

Group I, which received perioperative IN, consisted of 20 patients, including 8 women and 12 men aged 46 to 82 (median 68; Q1–Q3 63.5–71.5), with a median height of 171.5 cm (164.5–177.0) and weight of 78.2 kg (61.1–87.7). The median BMI was 25.4 (23.0–27.9). In this group of patients, unintentional weight loss in the last 6 months was10 kg (5.5–13.0), with a percentage of 12.6 (7.9–17). In 2 patients, no unintentional weight loss was noted.

Group II, consisting of 20 patients, did not receive IN. It consisted of 11 women and 8 men aged 47 to 85 (median 64.5; Q1–Q3 61.0–66.5), with a median height of 169 cm (161.5–175.0) and body weight of 69.8 kg (59.0–73.9). The median BMI was 23.1 (21.9–28.1). Unintentional weight loss in the last 3 months was 5.0 kg (0.0–8.5), with a percentage of 7.8 (0.0–13.1). There was no unintentional weight loss in 5 patients.

The value of the PA assessed on admission to the hospital in Group I was 5.0° (4.70°–5.85°), while in Group II, it was 5.1° (5.00°–6.25°).

The characteristics of the two groups are presented in Table 1.

In Group I receiving perioperative IN, in 12 (60%) patients, the tumor was located in the head of the pancreas, and in 8 (40%) patients in the body or tail of the pancreas. In Group II not receiving IN, there were 12 (60%) patients with a pancreatic head tumor and 8 (40%) with a pancreatic body/tail tumor.

**Figure 1:**
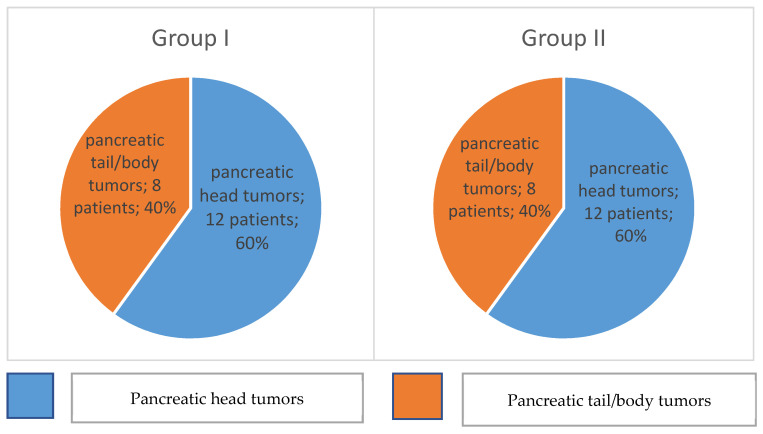
Location of the pancreatic tumor among the two groups.

The location of the tumor in the pancreas among the groups is shown in Figure 1.

### 3.2. Inflammation

CRP (Group I: 5.4 mg/dL; Group II: 5.0 mg/dL) and IL-1α (Group I: 11.1 pg/mL; Group II: 14.8 pg/mL) levels were similar in both groups on admission to the hospital. After 5 days of preoperative immunonutrition, no statistically significant changes were observed.

In Group II, which did not receive immunonutrition, a statistically significant increase in CRP (86.6 mg/dL; *p* < 0.02) and IL-1α (18.5 pg/mL; *p* < 0.03) levels was observed on the eighth day after surgery. The concentrations of CRP and IL-1α on admission to the hospital, after 5 days of preoperative IN, on the eighth day after surgery are shown in Table 2.

### 3.3. Phase Angle

In Group I, the PA on admission to the hospital was 5.0° (4.70–5.85). After 5 days of preoperative IN, a statistically significant (*p* < 0.02) increase in the phase angle to 5.35° (4.65–5.58) was observed. This parameter was also measured on days III and VIII. A statistically significant decrease (*p* < 0.001) in the PA to 4.65° (4.10–5.25) was observed on the third day. On the eighth day, the phase angle increased to 4.85° (4.30–5.45). On this day, no statistically significant difference was observed compared to the value of the PA on admission.

In Group II, the value of the PA on admission was 5.1° (5.00–6.25). On the third day after surgery, a statistically significant (*p* < 0.002) decrease in the value of the PA to 4.5° (3.75–5.36) was observed, while on the eighth day, despite the fact that the value of the PA slightly increased to 4.55° (4.0–5.1), it was still statistically significantly lower than on admission to the hospital (*p* < 0.008).

The above data indicate that the applied model of IN has a positive effect on the value of the PA. After the use of preoperative IN, the PA increased statistically significantly and, despite a statistically significant decrease on the third day, another increase was observed on the eighth day and no statistically significant difference was observed compared to the PA on admission, whereas in Group II, which did not receive IN, statistically significant decreases in the PA were observed on the third and eighth days.

PA on admission to the hospital after 5 days of IN in the first group, and then, on the third and eighth days in both groups are presented in Table 3.

### 3.4. Complications

In Group I, postoperative complications occurred in 55% (11) of patients, while in Group II, complications occurred in 65% of patients (13). Table 4 shows the observed postoperative complications classified according to the Clavien–Dindo classification.

**Figure 2:**
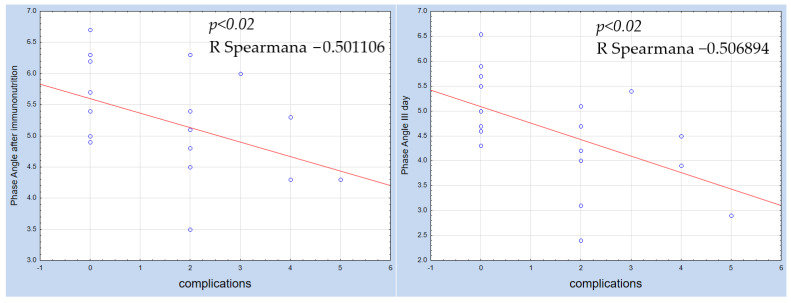
Correlation of the PA with complications after the applied model of IN and on the third postoperative day during early postoperative IN.

In Group I receiving perioperative IN, it was observed that the PA after 5 days of IN and on the third day after surgery during early postoperative IN negatively correlated (respectively, −0.501106, −0.506894), at a statistical significance of *p* < 0.02, with the incidence of complications. Correlation of the PA with complications after the applied model of IN and on the third postoperative day during early postoperative IN is shown in Figure 2. On the eighth postoperative day, no statistically significant correlation was observed.

In Group II, which did not receive IN, there were no statistically significant correlations between the PA and the occurrence of complications.

The correlations of PA in both groups with complications are presented in Table 5.

The above data indicate that the applied model of IN, by improving the PA, may reduce the frequency and severity of overall postoperative complications in patients operated on for pancreatic cancer.

## 4. Discussion

Pancreatic cancer is one of the deadliest cancers in the world. In most countries, there is an upward trend in morbidity and mortality due to pancreatic cancer. Currently, the primary treatment for pancreatic cancer is surgery. Malnutrition negatively impacts these patients in the postoperative period. Surgical trauma induces a metabolic response that activates its own energy sources to provide energy and building materials for the healing process. Metabolic changes lead to catabolism, worsening the abnormal nutritional status. The consequences of improper nutritional status and surgical trauma include disorders of the immune system. Pancreatic cancer induces an immunosuppressive inflammatory reaction. The result of these changes is an increased incidence of postoperative complications, such as infectious complications and impaired healing, not only of skin wounds but also of anastomosis with the pancreas [6,13,14].

In our own research, 23 patients (82.5%) experienced unintentional weight loss over the last 6 months, ranging from 1 kg (1.7%) to 30 kg (25.8%). The median was 8.0 kg, accounting for 9.9%, and in as many as 18 patients (45%), it exceeded 10%. According to the Global Leadership Initiative on Malnutrition (GLIM) criteria, malnutrition is diagnosed when unintentional weight loss is <5% in the last 6 months or >10% over 6 months [15]. However, only 10% (4) of patients had a body mass index below 20 kg/m^2^. Additionally, 47.5% (19) of patients were overweight (35% overweight, 12.5% obese I).

According to Hendifar AE et al., unintentional weight loss is observed in as many as 85% of patients with pancreatic cancer at the time of diagnosis. Almost 50% of patients with early-stage cancer and locally advanced disease experience preoperative unintentional weight loss, and 80% of them develop progressive unintentional weight loss after diagnosis [16]. Moreover, several studies confirm that patients with pancreatic cancer at the time of diagnosis have a normal or excessively high BMI index, despite experiencing significant unintentional weight loss, including the loss of visceral fat, skeletal muscle mass, and the development of sarcopenia (the loss of muscle mass and muscle strength) [17,18,19,20]. Currently, there are no approved treatments for weight loss associated with pancreatic cancer, and common methods such as the use of appetite stimulants do not yield clear benefits. Consequently, progressive weight loss remains one of the most challenging features to treat in pancreatic cancer [21]. The incorrect nutritional status accompanying pancreatic cancer and surgical treatment reduces immunity, leading to an increased frequency of postoperative complications. Therefore, it is crucial to identify patients at risk of malnutrition and those in the early stages of malnutrition. Improvement of treatment results is seen with the use of perioperative IN [6,14]. 

According to the 2012 ESPEN guidelines for perioperative care after pancreaticoduodenectomy, immunonutrition (IN) should be initiated 5–7 days before surgical treatment [22]. In 2017, the ESPEN guidelines for oncological patients strongly recommended the use of IN for patients with upper gastrointestinal tract cancer during the perioperative period, without specifying the dosage or timing of administration [23]. Similarly, the 2017 ESPEN guidelines for surgical patients recommended the use of compounds containing immunomodulating ingredients for malnourished patients undergoing extensive cancer surgery, either perioperatively or at least postoperatively [2]. The 2021 recommendations for oncological and surgical patients continue to endorse the use of IN during the perioperative or postoperative period, although specific recommendations regarding the dosage and duration of IN have not been established to date [7,8]. Thus, it has become crucial to establish an immunonutrition model for patients undergoing surgery for upper gastrointestinal tract cancer, which prompted us to develop our own model of perioperative immunonutrition in our research.

PA, an increasingly valued indicator, can be assessed through electrical impedance. Electrical bioimpedance, a measurement technique, employs an alternating current with a high frequency (enabling current passage through cell membranes) and low intensity to traverse the body. The result is derived from varying electrical resistances in distinct tissues. PA relies on the potential difference between extracellular and intracellular sides and is now recognized as an indicator of cellular health, offering an overall assessment at the cellular level. Numerous studies underscore its practical utility in oncology [24,25]. According to research by Małecka-Massalska et al., the appropriate PA value should fall between 5° and 7°, with values below 5° signifying abnormal nutritional status [24]. In our study, the PA was assessed in patients with pancreatic cancer who qualified for surgical treatment, and then, after 5 days of IN (Group I), on the third day during early postoperative IN and on the eighth postoperative day. Group II did not receive immunonutrition. Preoperatively, both groups exhibited a median PA within the correct range (Group I: 5.0° (4.70–5.85); Group II: 5.1° (5.00–6.25)). Interestingly, 27.5% (11) of the patients displayed a low PA. In Yasui-Yamada et al.’s study encompassing 501 patients with various gastrointestinal and hepatopancreatic ampulla tumors (155 patients with gastric cancer, 201 with colorectal cancer, 75 with liver cancer, 38 with biliary tract cancer, 32 with pancreatic cancer) they observed a median PA value of 5° in men (4.4–5.5°) and 4.4° in women (4.0–4.8°). In the pancreatic cancer subgroup, 10 (32.5%) patients had low PA values, 18 had normal values, and 4 had high values [25]. Tumas et al. assessed body composition in pancreatic cancer patients eligible for surgery, revealing that 39% of patients had a low median PA value [17].

Unfortunately, other studies show that the PA cutoff may vary depending on age, gender, BMI, and even race [26,27]. In the studies of Zhou S. et al., it was found that the PA cut-off point for patients with pancreatic head cancer should be 5.45°. PA values in malnourished patients were statistically significantly lower (*p* < 0.05) and correlated with the nutritional status of patients [28].

Interestingly, our own research showed a statistically significant increase in the PA after 5-day preoperative IN (5.35° (4.65–5.58)). In addition, despite a statistically significant decrease in the PA value on the third day after surgery during early postoperative IN, on the eighth day, the PA value increased and did not differ statistically significantly from the preoperative value. On the other hand, in Group II on days III and VIII, there were statistically significant decreases in the value of the PA. Interestingly, a statistically significant increase in CRP protein (86.6 mg/dL; *p* < 0.02 and IL-1α (18.5 pg/mL; *p* < 0.03)) was also observed on the eighth day after surgery in Group II. In Group I, an increase in CRP protein was also observed, but it was not statistically significant. IL-1α was slightly higher compared to the preoperative level. An important aspect seems to be that research by Tjomsland et al. showed that IL-1α was detected in most cases of pancreatic cancer, and high expression is associated with poor clinical outcomes [29,30,31].

This shows that the applied model of IN has a positive effect on the nutritional and inflammatory status of patients as well as the PA. In the available literature, there are no data evaluating the effect of IN on the PA in patients undergoing surgery due to pancreatic cancer.

Numerous studies have endeavored to establish a beneficial IN model for patients undergoing pancreatic cancer surgery. In a study by Gade et al., employing a preoperative IN model that included Impact Oral administered seven days before surgery to pancreatic cancer patients, no statistically significant effect was observed on postoperative complications or hospital stay duration [32]. Similarly, in Aida et al.’s study, the preoperative administration of IN (arginine, omega-3 fatty acids, nucleotides) for five days to patients eligible for pancreatoduodenectomy resulted in a reduction in the incidence of infectious complications and the severity of all complications [33]. Silvestri et al. employed a similar preoperative IN model, which led to a statistically lower rate of infectious complications (22.9% vs. 43.7%; *p* = 0.034) and shorter hospital stays (18.3 ± 6.8 days vs. 21.7 ± 8.3 days; *p* = 0.035) in the IN group [34]. Miyauchi et al. conducted a prospective randomized clinical trial comparing perioperative immunomodulatory nutrition with a preoperative model in pancreaticoduodenectomy patients. No additional benefit of perioperative immunomodulatory treatment over the preoperative model was observed [35]. Conversely, Suzuki et al. assessed perioperative IN in patients undergoing pancreatoduodenectomy and observed a reduced frequency of infectious complications [36]. Gianotti et al.’s 2000 study included pancreatic cancer patients eligible for pancreatoduodenectomy who received a postoperative standard formula containing immunomodulating components such as arginine, omega-3 fatty acids, and nucleotides. They found a lower incidence of complications (*p* = 0.005) and shorter hospital stays in the group receiving immunomodulatory components [37]. Yan et al.’s meta-analysis, analyzing 10 studies on various IN models in pancreatoduodenectomy-eligible patients, indicated that IN reduced hospital stay length and the incidence of infectious complications. However, no significant effects were observed on non-infectious complications, pancreatic fistula, delayed gastric emptying, mortality, C-reactive protein, or IL-6, suggesting the need for further research [38]. In our research, Group I, which received IN and had a higher PA, demonstrated a statistically significant negative correlation (*p* < 0.002; −0.501106) between PA and postoperative complications, with fewer complications observed in this group. In contrast, Group II exhibited no correlation between PA and complication occurrence. These findings suggest that the employed IN model enhances the postoperative course of pancreatic cancer patients. A lack of proper preparation of patients before surgery results in an increased risk of postoperative complications. Furthermore, the increase in PA in Group I indicates an improvement in patients’ nutritional status and immunity.

A limitation of our study is the small number of patients. Pancreatic cancer patients are a specific group. Only 20% of diagnosed patients have a resectable tumor. Pancreaticoduodenectomy is the largest surgery performed on the abdominal cavity. It is associated with a high risk of complications. Also, patient survival is low. According to various reports, the one-year survival rate is approximately 21% and the five-year survival rate is between 2% and 9% [39,40,41,42,43,44]. Our research has shown that there is a need to continue research on a larger group of patients, because our IN model improves the postoperative course.

## 5. Conclusions

This study used our own model of immunonutrition in patients undergoing surgery for pancreatic cancer. There are no studies in the literature assessing such a model of immunonutrition in patients with pancreatic cancer. There are also no reports in the literature on the assessment of the phase angle in patients after pancreatic cancer surgery who received immunonutrition. The applied model of perioperative IN improves the postoperative course of patients operated on due to pancreatic cancer. Fewer complications were observed in patients in the group receiving IN. Also, the PA value increased after the 5-day preoperative IN, and the use of perioperative IN improved the PA value on the eighth postoperative day compared to the group that did not receive IN. On this day, an increase in inflammatory parameters was also observed in the group that did not receive IN. In addition, PA correlated negatively with complications. The PA can be a useful tool to assess the effectiveness of the applied IN, and thus, to predict the occurrence of postoperative complications.

Therefore, there is a further need for studies on larger groups of patients.

## Figures and Tables

**Table 1:** Group characteristics.

	Group I				Group II			
	Median		Q1–Q3		Median		Q1–Q3	
Sex	women 8; men 12				women 11; men 9			
Age (years)	68.0		63.5–71.5		64.5		61.0–66.5	
Body weight (cm)	78.2		61.1–87.7		69.8		59.0–73.9	
Height (cm)	171.5		164.5–177.0		169.0		161.5–175.0	
Unintentional weight loss	kg	%	kg	%	kg	%	kg	%
	10.0	12.6	5.5–13.0	7.9–17	5.0	7.8	0.0–8.5	0.0–13.1
BMI (kg/m^2^)	25.4		23.0–27.9		23.1		21.9–28.1	
PA on admission to the hospital (°)	5.0		4.70–5.85		5.1		5.00–6.25	

**Table 2:** Concentrations of C-reactive protein and Interleukin-1 α on admission to the hospital, after 5 days of preoperative IN, on the eighth day after surgery.

		On Admission to the Hospital		After 5 Days of Preoperative IN		Eighth Day	
		CRP (mg/L)	IL-1α (pg/mL)	CRP (mg/L)	IL-1α (pg/mL)	CRP (mg/L)	IL-1α (pg/mL)
Group I	Median	5.4	11.1	8.3 *p* = 0.432	11.1 *p* = 0.600	68.4 *p* = 0.123	14.8 *p* = 0.179
	Q1–Q3	2.1–11.5	7.37–18.5	3.85–51.1	6.44–18.5	38.5–174.8	7.37–14.8
Group II	Median	5.0	14.8	-	-	86.6 *p* < 0.02	18.5 *p* < 0.03
	Q1–Q3	3.2–11.2	11.1–18.5	-	-	37.75–142.9	11.1–25.8

**Table 3:** Phase angle on admission after 5 days of preoperative IN in Group I, and then, on the third and eighth days after surgery in both groups.

	On Admission to the Hospital	After 5 Days of Preoperative IN	Third Day	Eighth Day
Group I	5.0° (4.70–5.85)	5.35° (4.65–5.58) *p* < 0.02	4.65° (4.10–5.25) *p* < 0.001	4.85° (4.30–5.45) *p* = 0.16
Group II	5.1° (5.00–6.25)	-	4.5° (3.75–5.36) *p* < 0.002	4.55° (4.0–5.1) *p* < 0.008

**Table 4:** Postoperative complications according to the Clavien–Dindo classification.

	No Complications	I	II	IIIa	IIIb	IVa	IVb	V
Group I	9	-	7	1	2	1	0	0
Group II	7	-	7	1	2	0	2	1

**Table 5:** Correlations of PA in both groups with complications.

	After 5 Days of Preoperative IN *p*-Value Spearman Correlations	Third Day *p*-Value Spearman Correlations	Eighth Day *p*-Value Spearman Correlations
Group I	5.35 (4.65–5.58) *p* < 0.02 −0.501106	4.65 (4.10–5.25) *p* < 0.02 −0.506894	4.85 (4.30–5.45) *p* = 0.364590 −0.214157
Group II	5.1 (5.00–6.25) *p* = 0.917630 −0.104881	4.5 (3.75–5.36) *p* = 0.687878 −0.095792	4.55 (4.0–5.1) *p* = 0.644497 −0.109942

## Data Availability

The raw data generated and analyzed in the current study are not publicly available due to the appropriate protection of patients’ personal information, but are available from the corresponding author on reasonable request.
